# Influence of Bovine Serum Albumin on the Antibacterial Activity of Endodontic Irrigants against *Enterococcus Faecalis*

**Published:** 2009-10-10

**Authors:** Sedigheh Khedmat, Marziyeh Aligholi, Samaneh Sadeghi

**Affiliations:** 1 *Department of Endodontics, Dental School, Dental Research Center, Tehran University of Medical Sciences, Tehran, Iran*; 2 *Department of Microbiology, School of Medicine, Tehran University of Medical Sciences, Tehran, Iran*; 3 *Dentist, Dental School, Tehran University of Medical Sciences, Tehran, Iran*

**Keywords:** Endodontic Irrigants, Antibacterial Activity, Bovine Serum Albumin

## Abstract

**INTRODUCTION:** It has been demonstrated that organic content of the root canals can influence the antimicrobial capability of chemical irrigants. The aim of this study was to evaluate the effect of bovine serum albumin (BSA), as an organic material, on the antimicrobial activity of several intracanal irrigants.

**MATERIALS AND METHODS:** Bactericidal activity of Ethylenediaminetetraacetic acid (EDTA) 17%, citric acid 10%, Sodium hypochlorite (NaOCl) 5.25%, Chlorhexidine 0.2% (CHX), Smear Clear and Cetrimide 0.5% were tested by means of dilution-neutralization method. Contact times were 10 and 30 seconds, 5, 10, 30, 60 minutes and 24 hours. First 950 λ of the medicament was mixed with 50 λ of the bacterial suspension in an Eppendorf test tube. The suspensions were thoroughly mixed. Sterile water served as negative controls. After each contact time, 100 λ of samples was transferred to the Eppendorf test tubes which contained neutralizers. After 5 minutes, 50 λ of serial dilutions were cultured on brain heart infusion agar and incubated in aerobic conditions. Then colonies were counted and reported as cfu/mL. In half of the samples, medicaments were suspended in BSA 0.5% 30 minutes before examination to assess its possible inhibitory effect on the antibacterial activity.

**RESULTS:** NaOCl 5.25%, Cetrimide 0.5% and Smear Clear showed bactericidal activity within seconds after the incubation. BSA had no inhibitory effect on bactericidal activity of these three medicaments. CHX took 5 and10 minutes to kill all bacterial cells in the absence and presence of BSA, respectively. Citric acid and EDTA showed the least antibacterial activity.

**CONCLUSION:** In this study, NaOCl 5.25%, Cetrimide 0.5% and Smear Clear were significantly more effective against* E. faecalis* than EDTA 17% and citric acid 10% in the presence and absence of BSA. Also, in the presence of BSA, bactericidal activity of CHX 0.2% against *E. faecalis* was significantly more than EDTA after 10 and 30 minutes of contact time. EDTA and citric acid showed the least bactericidal activity. [Iranian Endodontic Journal 2009;4(4):139-43]

## INTRODUCTION

The use of antimicrobial agents in conjunction with mechanical canal preparation has been shown to reduce bacterial load in the root canals (1,2). However, bacteria may still remain in the root canal system after chemo-mechanical canal preparations (2-5). *Enterococcus (E) faecalis* is a resistant against antibacterial activity of some endodontic medicaments (1,5) and are frequently found in apical periodontitis of previously root treated teeth (6). The most popular endodontic irrigant is sodium hypochlorite (NaOCl). It is a bactericidal agent and an effective solvent for vital and necrotic organic tissue (3,7). However, it has toxic effect on the periapical tissues (8) and has no effect on inorganic component of smear layer (9,10). For effective removal of the smear layer, combination of NaOCl and a chelating agent or acidic solution such as Ethylenediaminetetraacetic Acid (EDTA), Smear Clear and citric acid have been recommended (10-12). Several studies have shown that Chlorhexidine gluconate (CHX) is a potent antimicrobial agent (7,13) and has low toxic effects (14).

Some investigations have demonstrated that the organic content within the root canals could influence the antimicrobial capability of chemical irrigants (15-17). However, in Sassone *et al.* study, there was no marked difference in antimicrobial activity when Bovine Serum Albumin (BSA) was added to antimicrobial agents (18).

The aim of this study was to evaluate the effect of BSA as an organic material on the antimicrobial activity of EDTA 17%, citric acid 10%, NaOCl 5.25%, CHX 0.2 %, Smear Clear and Cetrimide bromide 0.5% against *E. faecalis*.

## MATERIALS AND METHODS

The irrigants tested were EDTA 17% (Pulpdent, Watertown, MA), citric acid 10% (Merck KGaA, Darmstadt, Germany), NaOCl 5.25% (Vista Dental Products, Racine, WI), CHX 0.2% (Consepsis, Ultradent, Inc., USA), Smear Clear (Sybron Endo, Orange, CA) and Cetrimide bromide 0.5% (Merck, Germany).

The neutralizers tested for EDTA, citric acid, NaOCl and CHX were designated as A, B, C and D respectively.

***Neutralizer A:*** 32.25 g Tween 80, 6.25 mL 40% sodium bisulfate, 3.922 g sodium thiosulfate pentahydrate, 2.525 g calcium chloride, diluted to 250 mL and adjusted to pH 7 and sterilized by filtration. One gram of lecithin was aseptically added (19).

***Neutralizer B:*** Mixture of 70 mL 0.2 M sodium bicarbonate with approximately 30 mL of 0.2 M sodium carbonate to reach a pH of 9.6; this was then sterilized by filtration (20).

***Neutralizer C:*** 5.0 g of Sodium thiosulfate; diluted to 100 mL and was sterilized by filtration (21).

***Neutralizer D:*** 3% Tween 80, 0.3% lecithin and 0.1% cysteine sterile deionized water was used as diluent for the neutralizing solution and as the control solution. The neutralizing and control solutions were adjusted to pH 7±0.2 with a solution of NaOH and were filtered using a 0.22 μm Millipore membrane filter (22).

General neutralizer included 30 g polysorbate 80, 30 g saponin, 1 g L. histidine, 3 g lecithin, 5 g sodium thiosulphate, diluents to 1 liter, sterilized by filtration was used for Smear Clear and Cetrimide bromide, because there was no specific Neutralizer for them (23). The diluents (tryptone salt) comprised 1 g tryptone, 3.5 g sodium chloride, and 1000 mL distilled water; the mixture was boiled to completely dissolve the ingredients. It was adjusted to a PH 7.2 and sterilized at 121˚C for 20 minutes.

*E. faecalis *strain ATCC 29212 (MAST, England) was cultured on brain heart infusion agar. Colonies were suspended in saline and adjusted to 3.0 Macfarland equivalent to 10^9^ cfu/mL. The suspension was used within 15 minutes.

Bactericidal activity of irrigants was tested according to BS-EN -1040: 2005 (23) by means of the dilution-neutralization method. The contact times were 10 and 30 seconds; 5, 10, 30, 60 minutes and 24 hours.

First 950 λ of the medicaments were mixed with 50 λ of the bacterial suspension in Eppendorf test tubes.

The suspensions were thoroughly mixed. Sterile water served as negative controls. After contact times, 100 λ of samples was transferred to the Eppendorf test tubes which contained Neutralizer. After 5 minutes serial dilution (10^-1^, 10^-2 ^and 10^-3^) were performed to calculate cfu/mL of bacteria.

50 λ of serial dilutions were cultured on brain heart infusion agar and incubated in aerobic condition and 37˚C.

After 24 hours, colonies were counted and reported as cfu/mL. All measurements were repeated five times and their mean value Cfu was analyzed.

The substance BSA was tested for its possible inhibitory effect on the antibacterial activity of the medicaments. Medicaments were suspended in 0.5% BSA for 30 minutes before examination. The rest of the tests were performed as previously described. Data were analyzed using Krauskal-Wallis, Dunn, and Mann-Whitney *U* tests (P<0.05).

## RESULTS

In this study, NaOCl 5.25%, Cetrimide 0.5% and Smear Clear showed bactericidal activity within seconds after the incubation without bovine serum albumin. There were no viable *E. faecalis* microbes in the 10-seconds samples. Also, as evident from figures, there was significant difference between the above three irrigants with EDTA, CHX and citric acid (P<0.001). In the 10 seconds interval samples without BSA, the antibacterial activity of NaOCl, Smear Clear and Cetrimide was significantly greater than citric acid and EDTA (P=0.009). After 30 seconds, antibacterial activity of EDTA was significantly less than NaOCl, Smear Clear and Cetrimide (P=0.005).

**Figure 1:**
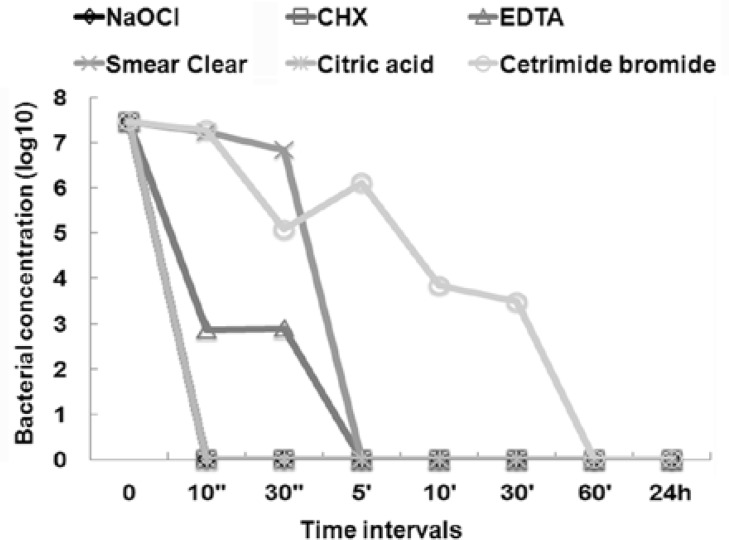
Eliminating* E. faecalis *with tested irrigants in the absence of BSA at different times.

Bovine serum albumin had no inhibitory effect on bacterial activity of NaOCl, Smear Clear and Cetrimide.

In the absence of BSA bactericidal activity of CHX 0.2% was detected 10 seconds after incubation, and overall 5 minutes was required for complete elimination of bacteria. However, BSA caused significant delay in the antibacterial effect of CHX 0.2% against *E. faecalis* after 10 seconds (P=0.009) and 5 minutes (P=0.005). A total of 10 minutes was required for eradication of bacterial cells by CHX 0.2% in the presence of BSA.

**Figure 2:**
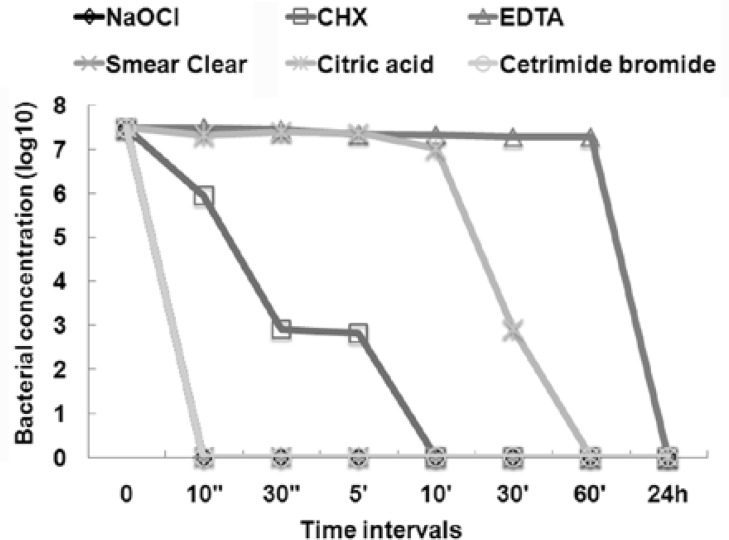
Eliminating *E. faecalis* with tested irrigants in the presence ofBSA at different times.

EDTA used without BSA was able to eradicate bacteria within 5 minutes like CHX; however BSA had the greatest inhibitory effect on EDTA, as bacterial elimination took 24 hours in its presence. Citric acid showed the least antibacterial activity when used alone and it took 60 minutes to reach CFU zero in the presence and absence of BSA (Figure 1), (Figure 2).

In presence of BSA, there was significant difference in antibacterial activity of NaOCl, Smear Clear and Cetrimide with EDTA after 10 seconds contact time (P=0.009). In the 30 seconds and 5 minutes intervals, antibacterial activity of NaOCl, Smear Clear and Cetrimide was significantly superior to EDTA (P=0.005) and citric acid (P=0.009).

There was significant difference between EDTA with all other irrigants except citric acid in the 10 and 30 minutes intervals (P<0.05).

## DISCUSSION

In present study, the dilution-neutralization technique recommended by the British standards institution was used to assay the antibacterial activity of tested irrigant on *E. faecalis *(23). Special neutralizers were used for all tested irrigants except for Smear Clear and Cetrimide, where general neutralizer was used due to lack of specific recommendation/special neutralizer.

The action of neutralizers is to eliminate the residual antimicrobial effect (carry-over effect) of the irrigants during processing of the samples; so antimicrobial activity can be determined correctly after specific time intervals without overestimation. *E. faecalis* was chosen as a mono-infection bacterium in root canals (6,24) as it has been suggested that endodontic irrigants must be effective against *E. faecalis* to be successful clinically (25). Also 0.5% concentration of BSA was added to half of the samples as an organic material to assess its influence on the antibacterial activity of various irrigants; several studies have demonstrated that the antibacterial activity of intracanal medicaments was reduced by the organic load within the root canals (15-17).

In this study, NaOCl 5.25%, Cetrimide 0.5% and Smear Clear eliminated *E. faecalis* bacteria rapidly. There was significant difference between these three irrigants with other tested irrigants in terms of antibacterial activity. Bactericidal activity of EDTA was significantly less than NaOCl 5.25%, Smear Clear, Cetrimide 0.05% and CHX 0.2%. The results for NaOCl confirmed the results of previous studies (18,21,25). The significant higher antibacterial activity of Smear Clear compared to EDTA was an interesting finding in this study, as it is composed of EDTA 17% solution with Cetrimide and special surfactant*.* This finding corroborated the result of Dunavant *et al.* (26) who reported antibacterial activity of Smear Clear against *E. faecalis* biofilm was more than CHX 2%, EDTA 17% and BioPure MTAD; however they showed that the difference between Smear Clear and EDTA was not significant. Antibacterial activity of Cetrimide alone as an endodontic irrigant has not been previously evaluated. The combination of CHX and Cetrimide has exhibited higher bactericidal activity compared to CHX alone (26). Superior antibacterial activity of Smear Clear compared to EDTA has related to the additional surfactant Cetrimide (26). Also, in the present study Cetrimide showed bactericidal activity within seconds after direct contact. Furthermore, the result of this study showed lower antibacterial activity for EDTA when compared to NaOCl, Smear Clear and CHX; concurring with Dunavant *et al.* study (26).

In present study, all *E. faecalis *cells were eliminated by CHX 0.2% after 5 minutes contact; however BSA delayed its antibacterial activity by 10 minutes. These results are similar to Portenier *et al.* (27) findings: no viable cells of two *E. faecalis *strains could be measured after 5 minutes of direct contact with CHX 0.2% and BSA inhibited its antibacterial activity. Citric acid was not able to abolish *E. faecalis* cells compelety after 5, 10 and 30 minutes intervals. This result is similar to the report by Krause *et al.* (21). In their study, citric acid 10% was not more effective than the saline control in the bovine tooth model infected with *E. faecalis* after 10 minutes of irrigation. According to our study, EDTA and citric acid showed less antibacterial activity than other irrigants. In the presence of BSA, citric acid required less time than EDTA to exert its action against *E. faecalis. *This difference may be influenced by the lower pH of the citric acid solution compared to EDTA that caused dissolution of BSA in the citric acid samples. However, the difference between antibacterial activities of these two medicaments against *E. faecalis* was not statistically significant. EDTA and citric acid have also been investigated as chelating agents for the smear layer removal (10,28-30). Some studies demonstrated similar action and efficiency in smear layer removal of these two medicaments (10,29,30).

In our method, only one microorganism was in close contact with irrigants. However, the clinical efficacy of endodontic irrigants should be considered with regards to complexity of root canal anatomy, polymicrobial nature of root canal infections and the presence of biofilms that may have reduced susceptibility to antimicrobial agents. Therefore, *in vitro* antibacterial effectiveness of irrigants may not accurately represent the* in vivo* condition.

## CONCLUSION

NaOCl 5.25%, Cetrimide 0.5% and Smear Clear were significantly more effective against* E. faecalis* compared to EDTA 17% and citric acid 10% in the presence and absence of BSA. Antibacterial activity of Cetrimide alone as an endodontic irrigant has not been evaluated; more studies are needed before its clinical use. In the presence of BSA, bactericidal activity of CHX 0.2% against *E. faecalis* was significantly more than EDTA after 10 and 30 minutes of contact. EDTA and citric acid showed the least bactericidal activity.
